# Decreased demand for olfactory periglomerular cells impacts on neural precursor cell viability in the rostral migratory stream

**DOI:** 10.1038/srep32203

**Published:** 2016-08-30

**Authors:** Anika Langenfurth, Song Gu, Verena Bautze, Caiyi Zhang, Julia E. Neumann, Ulrich Schüller, Kristin Stock, Susanne A. Wolf, Anna-Maria Maier, Giorgia Mastrella, Andrew Pak, Hongwei Cheng, Roland E. Kälin, Kenn Holmbeck, Jörg Strotmann, Helmut Kettenmann, Rainer Glass

**Affiliations:** 1 Cellular Neurosciences, Max Delbrück Centre for Molecular Medicine in the Helmholtz Association, Robert Rössle Str. 10, Berlin, Germany; 2 Department of Psychiatry and Psychotherapy/Neuropsychiatry, Charité - Universitätsmedizin Berlin, Charitéplatz 1, Berlin, Germany; 3 Neurosurgical Research, Ludwig Maximilians University Munich, Marchioninistr. 15, Munich, Germany; 4 Department of Neurosurgery, The First Affiliated Hospital of Anhui Medical University, Hefei, Anhui, China, 230022; 5 Institute for Physiology, University of Hohenheim, Garbenstr. 30, Hohenheim, Germany; 6 Center for Neuropathology, Ludwig Maximilians University Munich, Feodor-Lynen-Str. 23, Munich, Germany; 7 Institute of Neuropathology, University Medical Center, Hamburg-Eppendorf, Germany; 8 Research Institute Children’s Cancer Center, Hamburg, Germany; 9 Department of Pediatric Hematology and Oncology, University Medical Center Hamburg-Eppendorf, 20264 Hamburg, Germany; 10 Craniofacial and Skeletal Diseases Branch, NIDCR, National Institutes of Health (NIH), Bethesda, Maryland 20892-4380, USA

## Abstract

The subventricular zone (SVZ) provides a constant supply of new neurons to the olfactory bulb (OB). Different studies have investigated the role of olfactory sensory input to neural precursor cell (NPC) turnover in the SVZ but it was not addressed if a reduced demand specifically for periglomerular neurons impacts on NPC-traits in the rostral migratory stream (RMS). We here report that membrane type-1 matrix metalloproteinase (MT1-MMP) deficient mice have reduced complexity of the nasal turbinates, decreased sensory innervation of the OB, reduced numbers of olfactory glomeruli and reduced OB-size without alterations in SVZ neurogenesis. Large parts of the RMS were fully preserved in MT1-MMP-deficient mice, but we detected an increase in cell death-levels and a decrease in SVZ-derived neuroblasts in the distal RMS, as compared to controls. BrdU-tracking experiments showed that homing of NPCs specifically to the glomerular layer was reduced in MT1-MMP-deficient mice in contrast to controls while numbers of tracked cells remained equal in other OB-layers throughout all experimental groups. Altogether, our data show the demand for olfactory interneurons in the glomerular layer modulates cell turnover in the RMS, but has no impact on subventricular neurogenesis.

The adult mammalian brain contains two stem cell niches, the dentate gyrus in the hippocampus and the subventricular zone (SVZ) that is located along the lateral ventricles^1^. The SVZ consists of three proliferative cell types, which are summarized as neural precursor cells (NPCs). In particular, the SVZ contains *bona fide* stem cells (neural stem cells or type-B cells), transit amplifying cells (type-C cells) and immature cells that have committed to the neuronal (neuroblasts or type-A cells) or glial lineage^1^. In the adult murine brain, type-A cells constitutively migrate from the SVZ along a conserved migratory path, named the rostral migratory stream (RMS), into the olfactory bulb (OB)^1^,^2^. Astrocytes participate in maintaining the RMS by forming a tube-like structure around the migratory type-A cells and thus guide the neuroblasts to their final destination in the OB. In the RMS type-A cells migrate as cell-clusters, which is also referred to as chain migration^3^. Once type-A cells reach the OB, they radially migrate out from the RMS toward the granule cell layer^4^. The majority of neuroblasts differentiate into GABAergic granule neurons and form dendro-dendritic synapses^4^. A minority of subventricular type-A cells migrate into the glomerular layer and become GABAergic (and to a minor extent also dopaminergic or glutamatergic) periglomerular neurons, which form signaling-trajectories between neighboring glomeruli^5^. However, the acquisition of a periglomerular cell-fate appears to be controlled in the distal part of the RMS (proximal to the OB)^6^,^7^,^8^,^9^. In synopsis, NPCs from two different germinal zones in the olfactory system, namely the SVZ and the RMS, preferentially integrate into different neuronal networks in the OB, i.e. in the granule cell layer or in the glomerular layer.

Both NPCs in the SVZ and RMS provide a level of cellular plasticity for the olfactory system that likely is important for rodents to adapt to olfactory cues in the habitat. It was suggested that an additional level of complexity in regulating olfactory neuronal networks is reached by signalling pathways independently controlling the contribution of new granule-cells from the SVZ or new periglomerular neurons from the RMS^9^. Different paradigms for olfactory sensory deprivation were previously used to inspect the effect of olfactory input to NPC-turnover and to dissect differential regulation of NPCs in the SVZ and RMS, but different invasive techniques yielded divergent outcomes: In one study bulbectomy resulted in increased cell numbers within the RMS without affecting proliferation rates in the stem cell niche or in the RMS^10^; these data were interpreted that the OB is not necessary for maintaining proliferation in the SVZ or for directed migration in the RMS. On the contrary, other researchers reported that bulbectomy has profound effects specifically on stem cell-mediated neurogenesis in the SVZ^11^. Chemical lesions of the olfactory epithelium resulted in increased proliferation of slowly dividing cells in the RMS^9^, but odor-deprivation resulted in altered proliferation of fast dividing cells in the SVZ^12^. The main reason for the different outcome of these studies likely is the level of cell-death or inflammation induced by the different approaches since pathological stimuli alone can modify SVZ plasticity^13^. In this study we present a transgenic mouse model, which has reduced numbers of newly generated periglomerular neurons without any pathological side effects in the brain, like e.g. inflammatory reactions.

The number of sensory neurons, and hence the extent of sensory innervation of the OB, is limited e.g. by the size of the olfactory epithelium (i.e. a small olfactory epithelium can harbor fewer sensory neurons than a large epithelium). The surface of the olfactory epithelium is enlarged by convoluted structures, named turbinates, in the nasal cavity. The turbinates are formed by bone and cartilage and build the structural base of the olfactory epithelium^14^. The developmental morphogenesis of the turbinates depends on the gelatinolytic activity of membrane type-1 matrix metalloprotease (MT1-MMP) and MT1-MMP can contribute to the correct formation of the palate^14^. We have now discovered that MT1-MMP is of importance for the formation of nasal turbinates and that MT1-MMP deficient (MT1-MMP^−/−^) mice^15^ have largely reduced complexity of the turbinates, a concomitant reduction in olfactory epithelium, strongly reduced olfactory sensory innervation to the OB, reduced numbers of glomeruli and concomitantly decreased quantities of new periglomerular cells together with a profoundly reduced OB-size. Importantly, MT1-MMP^−/−^ animals have no developmental defects in the brain and no signs for inflammatory reactions; central findings from germline MT1-MMP knockouts were corroborated by us using a bone-specific model for MT1-MMP ablation. Hence, this model allows inspecting the effect of a reduced demand for new periglomerular neurons on the levels of neurogenesis in the SVZ and RMS under physiological conditions.

## Materials and Methods

### Animals

All experiments on live animals (mice) were carried out in accordance with the approved guidelines and regulations at the Max Delbrück Center for Molecular Medicine (MDC) and all experimental protocols were approved by the local authorities (Landesamt für Gesundheit und Soziales, LaGeSo, in Berlin): MT1-MMP^−/−^/GFAPcre^+^/ROSAbetaGAL^+^ and MT1-MMP^+/−^/GFAPcre^+^/ROSAbetaGAL^+^ as well as MT1-MMP^+/+^/GFAPcre^+^/ROSAbetaGAL^+^ litters; Osx-cre/MT1-MMP^FL/FL^ mice^16^, Osx-cre/MT1-MMP^FL/WT^ or Mt1-MMP^FL/FL^; MT1-MMP^−/−^/mOR256-17-GFP mice and MT1-MMP^+/−^/mOR256-17-GFP as well as MT1-MMP^+/+^/mOR256-17-GFP litters) were housed with a 12 h light/dark cycle and received food ad libitum.

*In situ* hybridization studies on wild-type mice were carried out on strain C57Bl6/J. Analyses of the projection pattern from olfactory sensory neurons expressing mOR256-17 were performed using our previously generated transgenic mouse strain which carries a targeted mutation of IRES-tauGFP at the mOR256-17 gene locus^17^. For tissue preparations, animals were anesthetised and sacrificed as approved by the local administrative authority.

### BrdU labelling

For tracing of neuroblasts with the thymidine analogue BrdU and for the detection of label-retaining cells in the SVZ (type-B cells) we injected mice (germline and conditional MT1-MMP deficient mice as well as controls) with an effective dose of 50 mg/kg BrdU (5-bromo-2-deoxyuridine, Sigma, Munich, Germany; at a concentration of 10 mg/ml BrdU in sterile 0.9% NaCl solution) i.p. 2x daily for three days; then mice were left untreated for 18 days; subsequently animals were sacrificed und prepared for immunolabelling studies. For tracing of type-C cells we administered a single BrdU-pulse 2 h prior to sacrifice and for labelling of type-A cells we applied a single BrdU injection followed by a 2 days chase period. Previously, in some (rare) instances a cell-viability compromising effect of BrdU was observed. We determined potential side effects of BrdU on the total number of dying subventricular NPCs by immunofluorescence co-labelling for active caspase-3 and BrdU and found that BrdU administration had no impact on NPC viability in any experimental group. For labelling of proliferating NPCs, animals received intra-peritoneal injections of 50 mg BrdU/kg body weight 2 h before euthanasia.

### Immunohistochemistry

All histological staining, immunofluorescence and immunohistochemistry labelling and subsequent stereological quantification was carried out as described previously^18^,^19^,^20^ (see also Supplementary information). Immunofluorescent triple labelling was carried out as described previously^21^ with the following primary antibodies: rat anti-BrdU (Biozol, Eching, Germany); rabbit anti-Ki67 (Novacastra Laboratories Ltd., Newcastle upon Tyne, UK); rabbit anti-Doublecortin (Santa Cruz Biotechnology, Heidelberg, Germany); mouse anti-PSA-NCAM and mouse anti-NeuN (Chemicon, Temecula, CA); goat anti-GFAP and mouse anti-p75NTR (R&D Systems, Wiesbaden, Germany); rabbit anti F4/80, mouse anti CD68, rabbit anti Mash1 and rabbit anti-S100ß (Abcam, Cambridge, UK); rabbit anti-MT1-MMP (Epitomics, Burlingame, CA); rabbit anti-Iba1 and goat anti Iba1 (WAKO, Neuss, Germany); rabbit anti-beta-Galactosidase (ICN Biomedical; Irvine; CA); mouse anti-calbindin (Sigma, Munich, Germany), mouse anti-calretinin (BD Bioscience), mouse anti-tyrosine-hydroxylase (Millipore, Darmstadt, Germany); cleaved caspase-3 (New England Biolabs, Frankfurt/M, Germany); mouse anti CD209 (Novus Biologicals; Wiesbaden; Germany); rabbit anti CD11c (BioRad, Munich, Germany); FITC-, RhodX-, or Cy5-conjugated secondary antibodies (all from Jackson ImmunoResearch Laboratories, West Grove, PA), Streptavidin Alexa Fluor555 (Invitrogen, Darmstadt, Germany).

### *In situ* hybridization of the olfactory epithelium

Digoxigenin-labelled antisense riboprobes were generated from partial cDNA clones in pGEM-T plasmids encoding mOR37A (NCBI accession number NM_019473.1, position 4-997), mOR256-17 (NM_008762.2, position 1-925), OMP (NM_011010.2, position 96-2062), Gap43 (BC_028288.1, position 27-832) and M72 (NM_030553.2, position 40-915) by using the T7/SP6 RNA transcription system (Roche Diagnostics, Mannheim, Germany) as recommended by the manufacturer.

*In situ* hybridization experiments were performed on 12 μm cryosections that were prepared from unfixed heads of young animals, sacrificed by decapitation and freshly embedded in ‘Tissue Freezing Medium’ (Leica Microsystems, Bensheim, Germany). The nose was sectioned using a CM3050S cryostat (Leica Microsystems) and the sections adhered to starfrost slides (Knittel Gläser, Braunschweig, Germany). Hybridization signals were visualized by using NBT (nitroblue tetrazolium) and BCIP (5-bromo-4-chloro-3-indolyl phosphate) in AP-buffer as substrates. Finally, the sections were rinsed with H_2_O and mounted in MOWIOL (33% glycerin, 13% polyvinylalcohol 4-88 (Sigma-Aldrich, Schnelldorf, Germany) in 130 mM Tris, pH 8.5).

### Fiber tracking using anti-GFP immunohistochemistry

To amplify the intrinsic fluorescence of GFP, immunohistochemistry using anti-GFP antibodies was performed. For this purpose the specimen were immersed for 30 minutes in fixative (4% paraformaldehyde in 150 mM phosphate buffer, pH 7.4, 4 °C) and kryoprotected by incubation in 30% sucrose in PBS overnight at 4 °C. For sectioning, all bones surrounding the olfactory bulb and the nasal turbinates were excised. Finally, the tissue was embedded in ‘Tissue Freezing Medium’ (Leica) and frozen on dry ice. 12 μm sections were generated and mounted onto microscope slides (Superfrost slides, Menzel, Braunschweig, Germany). Sections were air dried for 30 min and rinsed in PBS for 10 min at room temperature and subsequently incubated overnight at 4 °C with a rabbit-anti-GFP antibody (Invitrogen, Karlsruhe, Germany) diluted 1:800 in PBS/0.3% Triton X-100 containing 10% normal goat serum (NGS) (Dianova, Hamburg, Germany). After three rinses for 5 min in PBS, the bound primary antibodies were visualized by a secondary antibody conjugated with Alexa 488 (Invitrogen, Karlsruhe, Germany), diluted 1:600 in PBS/0.3% Triton X-100/10% NGS and incubated for 2 hours at room temperature. After washing three times for 5 min, the sections were counterstained with DAPI (1 μg/ml) (Sigma-Aldrich) for 3 min at room temperature, rinsed with H_2_O and mounted in MOWIOL.

### Microscopy and photography

Sections were analysed using a Zeiss Axiophot microscope (Carl Zeiss MicroImaging, Jena, Germany). Images were captured using a Zeiss Axiocam for transmitted light and a ‘Sensi-Cam’ CCD-camera (PCO-imaging, Kelheim, Germany) for fluorescent images.

### Quantitative analyses of the nasal cavity, mOR256-17 OSNs and glomeruli

For the quantification of nasal structures, images of every 10^th^ cross section along the anterior-posterior axis through the nasal cavity (20 images total) from wildtype and MT1-MMP^−/−^ animals were inspected using a 2.5 x objective and analysed using ImageJ software. Values from each individual were summed up to yield the epithelial surface. The GFP-labeled mOR256-17 expressing cells that were located on the nasal septum of the animals were counted at high magnification (20 x objective) on every 6^th^ coronal section (12 μm thick) along the rostro-caudal axis of the main olfactory epithelium. Statistical significance was determined by using the unpaired t-test in the Graphpad Software; significance was set at p < 0.05. For size measurements of mOR256-17 glomeruli, the AxioVision software (Zeiss, Jena, Germany; Release 4.6.3) was used. The area of the medial glomerulus on every coronal section (12 μm) through this structure was measured and the total area was calculated by summing up the cross-sectional values. The achieved value represents the mean size of the medial glomerulus including SD.

### TUNEL and Hoechst 33358 labelling

After completing immuno-histochemical staining for BrdU, Hoechst 33358 was applied at a concentration of 50 ng/ml in Tris buffered saline (TBS) for 15 min to the slides. Subsequently the slides were mounted and used for whole cell counts. TUNEL labelling for DNA 3′-strand breaks, which occur during apoptosis and necrosis, have essentially been performed as described^22^.

### Cell counting and unbiased stereology

Samples for quantification of proliferating or dying cells in the SVZ and RMS were taken from 4 mice or more per age and per time-point. We counted proliferating or TUNEL+ cells in 10 horizontal sections (with matching stereotactic coordinates in MT1-MMP deficient mice and controls). For cell-quantification in the SVZ we counted marker-positive cells in an area of 3–6 cellular layers from the ventricular lumen. Numbers of labelled cells were counted by an unbiased approach using a modified optical fractionator procedure (StereoInvestigator, MicroBrightField Inc., VT, USA), which was previously described^21^. For the determination of whole cell numbers in the SVZ we used every 6^th^ brain section and a sampling method provided by the optical fractionator in the StereoInvestigator program, after Hoechst 33358 labelling of samples corresponding to those described above (coefficient of error, Gundersen ≤0.08). For confocal cell counting we used two defined fields in the SVZ^23^ at a defined distance of 2.3 mm, 2.6 mm, 2.9 mm and 3.2 mm from the dural surface (stereotactic coordinates were retrieved from a mouse brain atlas: http://mouse.brain-map.org/static/atlas; 4 animals per co-labelling experiment were used).

Quantification of numbers and size of olfactory glomeruli and of glomerular interneuron subtypes was performed in 4 mice per group (wild-type or MT1-MMP deficient animals at postnatal day 20; P20). Horizontal sections through the olfactory bulb were obtained at defined anatomical layers (at a distance of 1.5, 2.0, 2.5, 3.0, 3.5 and 4.0 mm from the dural surface; as measured in the dorso-ventral axis) in all groups. The morphology of the olfactory glomeruli was visualised by Haematoxilin and Eosin histochemical staining and the size of individual glomeruli was measured with the StereoInvestigator software. To quantify the numbers of interneuron subtypes we used again brain section from defined anatomical layers (as described above) immunostained for interneuron markers and quantified the neuronal subtypes in the glomerular layer (which can be unequivocally identified in immunohistochemical sections). Interneuron numbers were counted using the modified optical fractionator procedure described above and by others^21^.

The RMS was investigated after generating sagittal sections of mouse brains (4 mice per group from MT1-MMP-wild-type or-deficient animals at postnatal day 20; P20). Immunohistochemical stainings were performed with slices in an anatomical plane that was between 0.7 and 0.8 mm lateral from the bregma.

### Confocal microscopy

For confocal microscopy (TCS SP5 microscope, Leica, Wetzlar, Germany) we used appropriate gain and black level settings (determined on control tissues stained with secondary antibodies alone). All confocal images for marker colocalisation studies were taken with a 40x or a 63x magnification objective and were recorded as single images or image stacks, images were overlayed using Photoshop-CS software (Adobe, San Jose, CA, USA) and 3D reconstructions were obtained with Volocity software (PerkinElmer, MA, USA).

### Cell culture

Neurospheres were gained from micro-dissected and dissociated SVZ of P14 MT1-MMP wildtype and knockout mice, and were propagated in NB/B27 medium containing neurobasal medium (NB, Invitrogen, Karlsruhe, Germany), supplement (B27, Invitrogen), growth factors (20 ng/ml EGF and FGF; from PeproTech, Hamburg, Germany) and additives (0.2 mM glutamine, 100 U/ml penicillin, and 100 μg/ml streptomycin; from Sigma).

### Limiting dilution assay

Neurospheres were dissociated and plated in 200 μl NB/B27 medium per well in 96 well-plates. Cells were plated at following densities: 2000, 1500, 1000, 500, 200, 100, 50 and 10 cells/well. Neurosphere formation was evaluated after 7 days and the percentage of wells without neurosphere was plotted against the cell number and the x-intercept values were calculated.

### BrdU/Proliferation assay

For the BrdU incorporation assay, the BrdU Cell Proliferation Assay (QIA58, Calbiochem, Darmstadt, Germany) was used according to the manufacturers instructions. 3000 cells/well were plated on poly-L-ornithine/laminin-coated 96well plates and BrdU was added for 24 h.

To monitor proliferation, neural precursor cells were cultured as neurospheres plating 500 000 cells per 10 cm dish/passage. The resulting cell number was determined for each passage and the proliferation factor was calculated.

### Statistical analysis

Bar diagrams are shown as mean values ± standard deviation of the mean. Comparisons among the groups were performed with the Student’s t test.

## Results

### MT1-MMP-deficiency results in reduced sensory innervation of the OB

**Figure 1:**
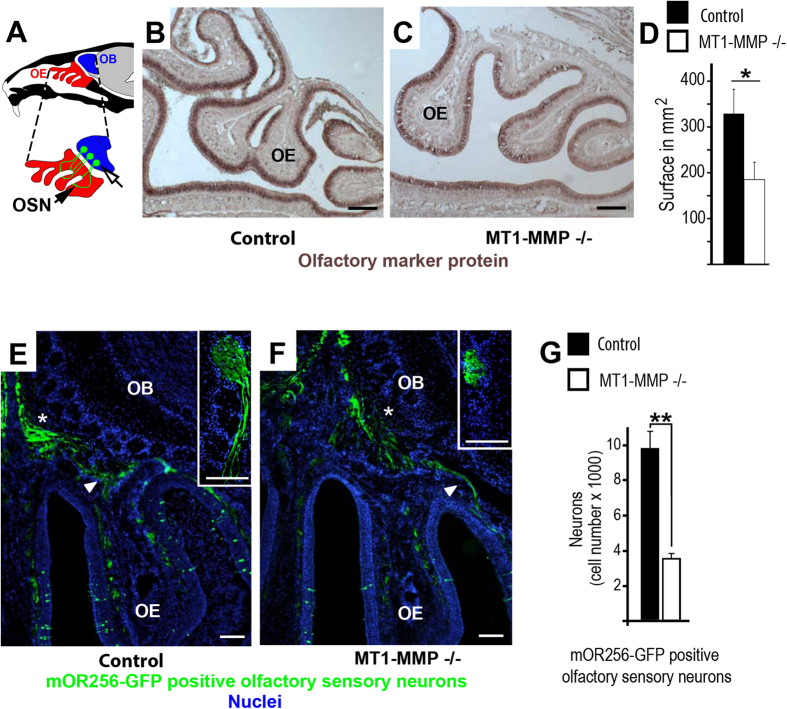
MT1-MMP deficiency affects the structure of olfactory turbinates and the level of olfactory bulb sensory innervation. (**A**) Overview on the olfactory epithelium (OE, foldings are termed turbinates) and olfactory bulb (OB); enlarged part shows olfactory sensory neurons and glomeruli; white area between olfactory epithelium and olfactory bulb is the cribriform plate; olfactory sensory neurons are abbreviated as OSN; the black and white arrow indicates olfactory glomeruli. (**B**,**C**) Turbinates in MT1-MMP^−/−^ were less complex than in controls, the olfactory epithelium (OE) was labelled with the olfactory marker protein and is visible in dark brown colour. (**D**) The surface area of the nasal turbinates is strongly reduced in MT1-MMP^−/−^ mice (white bar) as compared to wildtype controls (black bar; p = 0.0108) (**E**,**F**) Nasal cavity and anterior olfactory bulb of olfactory receptor mOR256-17 reporter mice (mOR256-17-GFP crossed with MT1-MMP^−/−^ or control): olfactory sensory neurons (arrowhead) pass through the cribriform plate (asterisk) and into a defined glomerulus (inserts in **D,E**); Nuclei are labeled with DAPI in blue. (**G**) The number of mOR256-17-GFP^+^ neurons was reduced in MT1-MMP^−/−^ mice. Scale bars: 200 μm in (**B**,**C**). 100 μm in (**E**,**F**); 100 μm in inserts (**E,F**); statistical significance: *represents p < 0.05; **represents p < 0.005.

The turnover of type-II collagen by MT1-MMP affects cranial development^15^. Type-II collagen is abundant in the developing nasal cavity^24^ and our present data show that MT1-MMP largely contributes to the formation of convoluted osteochondral structures within the nasal cavity referred to as the nasal turbinates. The olfactory mucosa that lines the turbinates contains olfactory sensory neurons which express odorant receptors and send axonal trajectories towards the OB^25^; for a schematic overview on the anatomy of the nasal cavity and the olfactory epithelium-derived sensory innervation of the OB see Fig. 1A. The olfactory marker protein (OMP) is a cytoplasmic protein expressed in mature olfactory sensory neurons and an established marker for neurons connecting the peripheral sensory epithelium with olfactory neuronal network within the OB^26^. *In situ* hybridization for OMP was used to study the anatomy of the nasal turbinates and the overlaying olfactory epithelium in wild-type and MT1-MMP^−/−^ animals (Fig. 1B,C). The folding of nasal turbinates was reduced in MT1-MMP^−/−^ mice (Fig. 1C) thereby decreasing olfactory epithelium surface as compared to controls (Fig. 1B). To obtain a quantitative measure of this observation, the epithelial surface lining the nasal turbinates was measured; we calculated an olfactory epithelial surface of 329.019 ± 53.14 mm^2^ in wildtype and 185.2 ± 37.9 mm^2^ in MT1-MMP^−/−^ mice (Fig. 1D).

To access the central nervous system (CNS) olfactory sensory neurons need to traverse a specialized part of the skull, the so-called cribriform plate. In order to investigate if MT1-MMP also impacts on the formation of osseous structures which allow neuronal trajectories to access the brain we traced distinct olfactory sensory neurons from the nasal cavity into the olfactory bulb using mOR256-17-GFP mice^17^ crossbred with MT1-MMP^−/−^ or wild-type controls (n = 4; Fig. 1E,F). The mOR256-17-GFP olfactory sensory neurons traversed the cribriform plate and coalesced into glomeruli as previously described^17^,^27^ but the number of olfactory sensory neurons was strongly reduced in MT1-MMP^−/−^ compared to controls (inserts in Fig. 1E–G). Overall, these data suggest that in MT1-MMP^−/−^ mice, as compared to control animals, a reduced complexity in the folding of the nasal turbinates resulted in reduced surface of the olfactory epithelium, fewer olfactory sensory neurons and decreased sensory innervation of the olfactory bulb.

MT1-MMP deficiency did not affect olfactory epithelium and olfactory sensory neuron development as evidenced by higher magnification micrographs from *in situ* hybridizations for OMP and for GAP43 (an mRNA expressed in immature sensory neurons). The correct anatomical layering for immature and mature sensory neurons was maintained in both MT1-MMP-knockouts and wild-type controls. Also we investigated the spatial organization of distinct olfactory sensory neurons within the olfactory epithelium in MT1-MMP^−/−^ and control mice. *In situ* hybridizations for the odorant receptors M72, mOR256-17 or mOR37A revealed a localization of these receptors within the olfactory epithelium consistent with previous reports^27^ (Supplemental Fig. 1B). Importantly, brain structures and cortical layers developed normally in both MT1-MMP^−/−^ mice and controls (Supplemental Fig. 2). Likewise, tracing of neural stem cell progeny from embryonic day 13.5 to adulthood revealed no differences in brain development of both strains (Supplemental Fig. 3). Altogether, these additional controls show that MT1-MMP-loss has large consequences specifically for bone formation and strongly diminishes the complex folding of turbinates while neurodevelopmental processes remain intact.

### MT1-MMP deficient mice have reduced OB-size and less olfactory glomeruli

**Figure 2:**
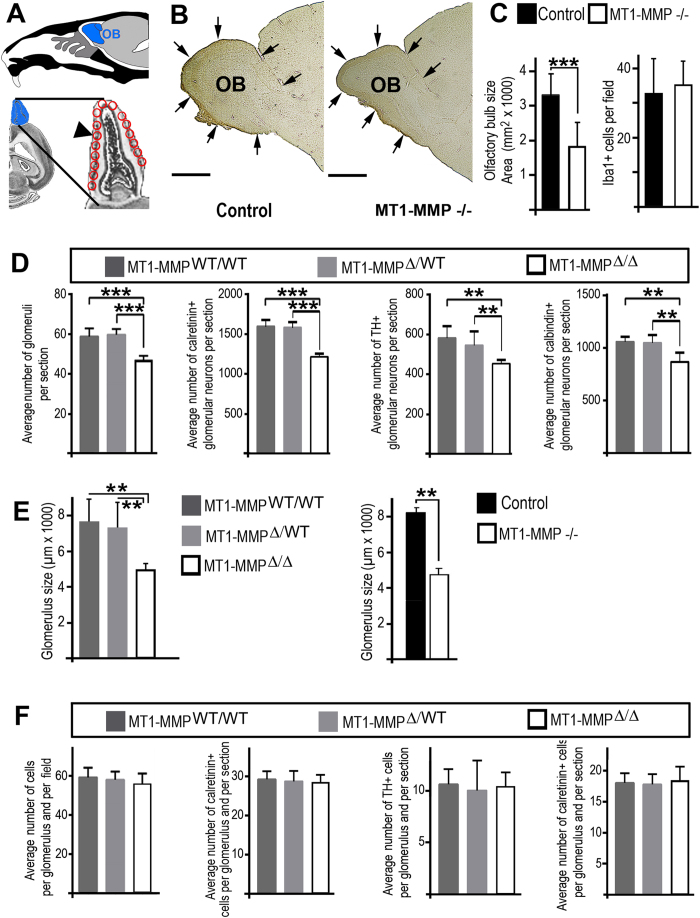
MT1-MMP deficiency affects the size of the olfactory bulb. (**A**) A schematic drawing shows the position of the frontal forebrain and olfactory bulb (OB) in relation to the nasal cavity (upper part); lower part: a horizontal section through the OB (blue structure) is magnified and the position of the olfactory glomeruli (red circles) is highlighted (arrowhead). (**B**) Phase contrast images of a sagittal section through the OB in MT1-MMP^−/−^ and controls. (**C**) Olfactory bulb size is largely reduced in MT1-MMP^−/−^ at P20; a reduction in OB-size is not associated with increased numbers for myeloid cells (Iba1+ cells) in the OB. (**D**) Histochemical staining and subsequent cell-counting were used to quantify the total number of cells per olfactory glomerulus in conditional MT1-MMP knockouts (MT1-MMP^Δ/Δ^) or controls (MT1-MMP^Δ/WT^, Mt1-MMP^FL/FL^); immunohistochemical staining for calretinin, tyrosine-hydroxylase (TH) or calbindin was followed by cell-counting to quantify numbers of interneuron subtypes in the glomerular cell layer. (**E**) Histochemical staining and morphometry were used to quantify the size of glomeruli in all mouse models. (**F**) Histochemistry or immunohistochemistry and cell-counting were performed to quantify the number of cells respectively the number of different interneuron in the glomerular cell layer. Scale bars: 1 mm in (**B**,**C**); statistical significance: ** represents p < 0.005; *** represents p < 0.001.

Next, we investigated if the reduced sensory innervation of the OB in MT1-MMP knockouts had consequences for the overall anatomy of the OB and for establishing neuronal networks processing olfactory information in the CNS; for a schematic overview see Fig. 2A. Strikingly, the size of the olfactory bulb at postnatal day 20 (P20) was significantly reduced in MT1-MMP^−/−^ mice (Fig. 2B) as compared to controls (Fig. 2C; statistical significance p = 0.00018). Maturation of the OB and the olfactory neuronal circuitry is a dynamic process that extents throughout postnatal development^28^. Therefore, we compared OB size in MT1-MMP-deficient animals and wild-type controls also at an earlier time-point after birth and found that reduced OB size was also detectable at P10 (statistical significance p = 0.04). Gross and fine anatomical inspection of the OB was repeated with an additional mouse model allowing the ablation of MT1-MMP specifically in bone-forming osteoblasts^16^. Osx-cre/MT1-MMP^FL/FL^ mice, which express cre-recombinase under control of the osteoblast-specific promoter element for the osterix gene and thereby recombine (ablate) loxP-flanked sites of the MT1-MMP gene (in the following abbreviated as MT1-MMP^Δ/Δ^), were compared to relevant controls (Osx-cre/MT1-MMP^FL/WT^, abbreviated as MT1-MMP^Δ/WT^, or cre-deficient MT1-MMP^FL/FL^). Again, a large and statistically significant reduction in OB size was observed in MT1-MMP deficient animals (MT1-MMP^Δ/Δ^) as compared to controls (MT1-MMP^Δ/WT^, p = 0.0026; MT1-MMP^FL/FL^, p = 0.00041; not shown). The conditional MT1-MMP knockout model and corresponding controls were also used to investigate potential neuroinflammatory reactions in our mouse strains. We inspected the myeloid cell morphology and marker expression (Iba1, CD68, CD11c or CD209) as well as lymphoid cell density (as summarized in Supplemental Fig. 4) in control and MT1-MMP deficient animals. We did not detect any signs for brain inflammation in our MT1-MMP^Δ/Δ^ mice or controls. Of note, MT1-MMP knockout-induced reduction in OB-size was not accompanied by alterations in myeloid cell number either (Fig. 2C).

Glomeruli of the OB are neuronal-activity dependent structures, which relay the sensory olfactory information from the olfactory epithelium to higher brain centres^29^. To uncover consequences of reduced OB-size in (conditional) MT1-MMP deficient mice we quantified the number and cellular composition of olfactory glomeruli. Therefore, we performed immunohistochemical stainings for the main subtypes of periglomerular neurons, which express tyrosine-hydroxylase (TH), calbindin or calretinin^30^,^31^, and quantified these cell-types (Fig. 2D). Furthermore we used histochemical stainings, as previously described by others^31^, to visualize glomerular structures and quantified cell-numbers per glomerulus. Consistent with the large reduction in OB-size in MT1-MMP deficient animals we observed an overall reduction in the number of olfactory glomeruli, as compared to controls (Fig. 2D). The numbers of all major subtypes of periglomerular neurons were also reduced in MT1-MMP^Δ/Δ^ as compared to MT1-MMP^Δ/WT^ or MT1-MMP^FL/FL^ mice (Fig. 2D). In addition, we found that the average size of olfactory glomeruli was smaller in MT1-MMP-deficient mice (conditional or germline knockouts) compared to controls (Fig. 2E), while cell-numbers per glomerulus or the number of interneurons per glomerulus remained constant (Fig. 2F). Such effects on the glomerular size were observed previously and interpreted to result from reduced complexity of dendritic structures in the glomeruli as well as reduced cell-sizes in the neuronal network which both point towards reduced neuronal network activity^32^,^33^. Altogether, mice lacking MT1-MMP^−/−^ expression (by MT1-MMP ablation specifically in the bone or after systemic knockout) had reduced sensory innervation to the olfactory bulb, smaller glomeruli, reduced numbers of all major types of periglomerular neurons and smaller olfactory bulbs compared to control littermates.

### Subventricular neurogenesis is not affected by a reduced demand for new olfactory interneurons

**Figure 3:**
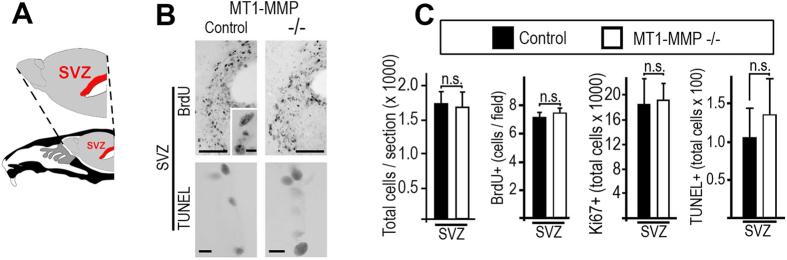
MT1-MMP deficiency has no effect on subventricular neurogenesis. (**A**) Scheme indicating the anatomical position of the subventricular zone (SVZ) in a sagittal brain section. (**B**,**C**) Immunohistochemistry for BrdU (labelled cells are magnified and shown in the insert; a 2 h BrdU-pulse was applied) in the SVZ; labelling for DNA strand breaks (TUNEL) indicating cell-death in the SVZ; representative staining for proliferative and dying subventricular cells in MT1-MMP^−/−^ mice and controls are shown. (**C**) Quantification of total cell numbers for BrdU^+^, Ki67^+^ and TUNEL^+^ cells in the SVZ of MT1-MMP knockouts and controls. Scale bars: 100 μm (BrdU) and 5 μm (for insert in BrdU), 10 μm (TUNEL); statistical significance: ***represents p < 0.001; *represents p < 0.05.

During a postnatal period the rodent brain generates large numbers of NPCs (10,000 to 30,000 cells per day^34^) which can differentiate into olfactory interneurons and thereby provide the means for cellular plasticity in the OB. These olfactory interneurons origin from the SVZ (for a schematic overview see Fig. 3A) and reach the OB via the RMS. To investigate if the reduced numbers of olfactory interneurons in MT1-MMP-deficient mice provide a signal for a reduced demand of new NPCs towards the SVZ we studied the level of subventricular neurogenesis in germline and conditional MT1-MMP knockouts as compared to controls. In particular, we inspected the morphology, proliferation and cell death levels of NPCs in the SVZ and RMS (Fig. 3B,C). We counted bromodeoxyuridine (BrdU) labeled NPCs in the SVZ and found that the average number of proliferating precursor cells per counting unit was not different in MT1-MMP^−/−^ when compared to controls (Fig. 3C). Also, a detailed analysis of the SVZ cell-composition in conditional MT1-MMP-knockouts and controls, using a combination of different BrdU labeling paradigms and cell-type specific markers, showed that there are no differences between MT1-MMP deficient mice and controls (Supplemental Fig. 5). First, we inspected the number of proliferating subventricular cells in MT1-MMP^Δ/Δ^ and control mice by repetitive BrdU application followed by a prolonged chase period. The population of label-retaining cells expressed the type-B cell markers GFAP and nestin and type-B cell numbers were equal in both mouse lines. Secondly, we administered a single BrdU-pulse given 2h prior to sacrifice (a short pulse of thymidine and analogues preferentially marks subventricular type-C cells^35^) and observed the BrdU-uptake in Mash1-positive type-C subventricular cells. Importantly, MT1-MMP deficient mice and controls harboured similar numbers of cycling type-C cells. We furthermore applied a single BrdU pulse followed by a 2 days chase-period to both mouse lines. Now we inspected the number of type-A cells (marked by PSA-NCAM expression) taking up the thymidine analogue and again observed no changes between MT1-MMP^Δ/Δ^ mice and controls (all data are presented in Supplemental Fig. 5). Since the different subtypes of OB interneurons (expressing TH, calbindin or calretinin) emanate from different regions of the SVZ^30^ we also looked out for regional heterogeneity in SVZ proliferation and did not observe any signs for altered cell-division levels in MT1-MMP^Δ/Δ^, MT1-MMP^Δ/WT^ or MT1-MMP^FL/FL^ animals. In addition we found that NPCs cultivated from P14 SVZ of MT1-MMP^−/−^ had similar doubling times, sphere formation capacity and BrdU retention levels as well as an equal propensity to differentiate into the neuronal lineage when compared to controls (Supplemental Fig. 6). Overall, our detailed analysis of subventricular cell-types, proliferation-rates and cell death levels shows that NPC-differentiation and -turnover are not altered by MT1-MMP ablation.

### NPCs-numbers are adapted to the demand for new periglomerular neurons within the RMS

**Figure 4:**
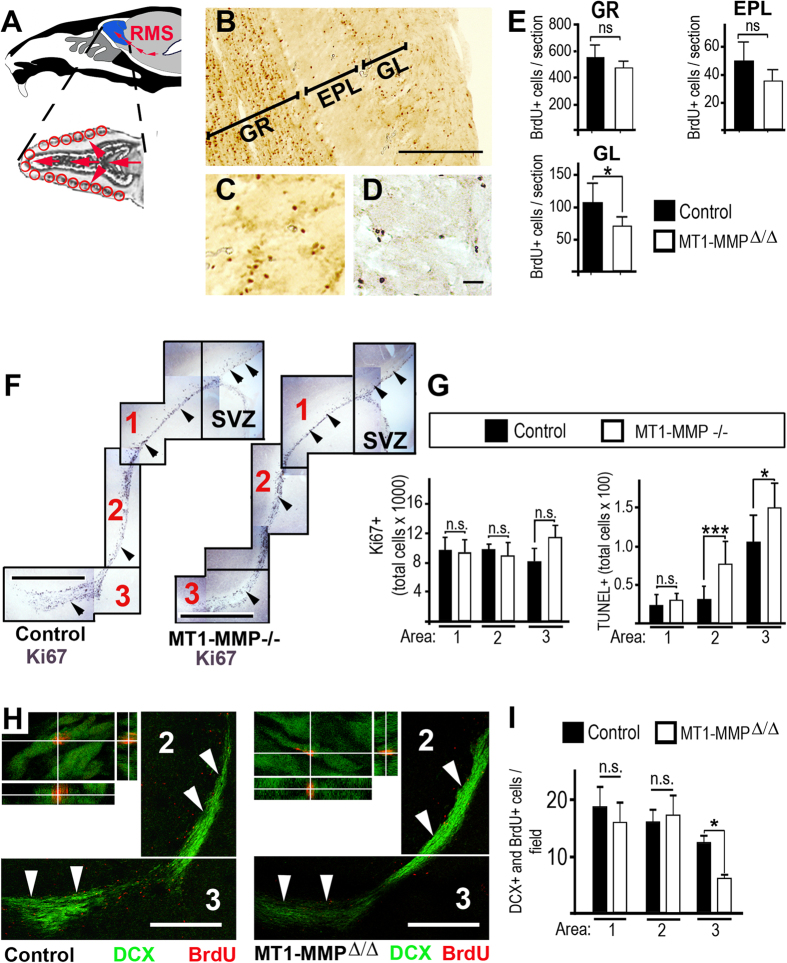
Reduced numbers of new periglomerular cells and increased cell-death levels in the RMS of MT1-MMP deficient mice. (**A**) Scheme indicating the localization of the RMS in a sagittal brain section and in a magnified horizontal section of the migratory path in the OB. (**B)** Immunohistochemistry for BrdU was performed on a horizontal OB-section: granule cell-layer (GR), external plexiform layer (EPL), glomerular layer (GL). (**C**,**D**) Representative staining for BrdU. (**E**) The number of BrdU+ cells (labelling protocol as described in **B**) was quantified in distinct layers of the olfactory bulb using horizontal sections of identical stereotactic coordinates in MT1-MMP^Δ/Δ^ or controls (summarized from MT1-MMP^Δ/WT^ and MT1-MMP^FL/FL^ experimental groups, which gave highly similar results). (**F**) Composite pictures showing representative immunolabelling for the proliferation marker Ki67 in the subventricular zone (SVZ) and rostral extension of the SVZ plus rostral migratory stream (divided into the parts 1, 2 and 3) of MT1-MMP^−/−^ and control mice. (**G**) Quantification of Ki67^+^ and TUNEL^+^ cells throughout the anatomical areas highlighted in F as 1, 2 and 3; note that cell-death levels in the RMS (area 2 or 3) are robustly increased in MT1-MMP deficient mice as compared to controls while proliferation-rates remain unchanged. (**H**) MT1-MMP^Δ/Δ^ mice or controls received repetitive BrdU-pulse followed by an extended chase period (for details see text) and sagittal brain sections were subsequently immunolabelled for DCX (green) and BrdU (red). Overviews on parts 2 and 3 of the RMS (indicated by arrowheads) are provided. Cells in the RMS were inspected by confocal microscopy and analysed for coexpression of markers (crosshair images); single optical sections and corresponding immunofluorescence over 4 μm in Z-orientation are presented; note that BrdU+/DCX+ cells can be clearly identified. (**I**) The number of DCX and BrdU colabelled cells was quantified in the proximal (area-1), intermediate (area-2) and distal (area-3) part of the RMS in MT1-MMP deficient and control mice. Scale bars: 250 μm in (**B**), 25 μm in (**C,D**) 1 mm in (**F**) and 500 μm in (**H**); statistical significance: n.s. indicates no statistical significance; *represents p < 0.05; ***represents p < 0.001.

Our data on the cellular composition of the OB indicated that mice lacking MT1-MMP have significantly lower levels of all three major types of OB-interneurons. This implies a reduced demand for new SVZ-derived cells, but our inspection of subventricular NPC-turnover revealed no differences in MT1-MMP deficient models and controls. We tracked BrdU-labelled cells (the labelling protocol comprised 2 pulses of BrdU over a period of three days followed by an 18 days chase period) in the OB and quantified the number of BrdU label-retaining cells in the glomerular layer, the external plexiform layer and the granule cell-layer (see Fig. 4B through E). Strikingly, we found that in MT-1MMP deficient mice (as compared to controls) the number of newly generated cells was statistically significantly reduced only within the glomerular layer, but not in the other layers of the OB (Fig. 4E). This showed that the reduced number of olfactory glomeruli, which we observed in germline- and conditional-models for MT1-MMP ablation, resulted in a specific reduction of new periglomerular neurons but did not cause significant deficits in the number of newly born interneurons in other OB-layers. Next, we inspected the rates of cell-proliferation and -death within the RMS of MT1-MMP-ablated and control mice. To provide a detailed view on the levels of dividing or dying cells in this region (the migratory axis from SVZ to OB can be up to 5 mm long in mice) quantification was performed in three anatomically distinct parts of the RMS (representative examples for the RMS in MT1-MMP-deficent and control mice are given in Fig. 4F). Most parts of the RMS (in Fig. 4F indicated as 1 and 2) of MT1-MMP^−/−^ mice did not exhibit any morphological differences to controls. Also the proliferation and cell death levels in RMS1 (proximal to the SVZ) were unchanged (Fig. 4G). However, we detected an increase in the number of TUNEL^+^ cells in RMS2 and RMS3 (distal from the SVZ) in MT1-MMP^−/−^ compared to controls (Fig. 4G). These TUNEL^+^ cells were confined to the RMS and we detected only very few TUNEL^+^ cells in other parts of the brain including the OB in both MT1-MMP^−/−^ and controls. These data suggested that wild-type and MT1-MMP^−/−^ mice generate equal numbers of neural progenitors but that in MT1-MMP deficient mice (in contrast to controls) a substantial part of progenitor cells dies in the distal RMS and therefore does not reach the OB. To substantiate this notion and to corroborate our findings from the MT1-MMP-knockout model we performed a BrdU-tracing experiment in MT1-MMP^Δ/Δ^ and control mice. Therefore, animals received repetitive BrdU pulse (2 i.p. injections of BrdU per day over a time-course of 3 days) followed by an 18 days chase period. With this labelling paradigm BrdU is incorporated in SVZ cells and labelled type-A cells which migrate along the RMS towards the OB. Since there are no differences in cell-division rates between MT1-MMP deficient or control mice in NPCs along the SVZ or RMS (see Figs 3B,C and 4F,G and Supplementary Fig. 5) the label is retained equally in traced cells of both mouse strains. Neural progenitor cells migrating along the RMS were identified by immunofluorescence for doublecortin (DCX) and analysed for BrdU incorporation (see Fig. 4H) in all three defined parts of the RMS (as previously outlined for Fig. 3F). We quantified the number of DCX and BrdU colabelled cells along the RMS and found that a substantially reduced number of SVZ-derived neural progenitors is present in the distal RMS of MT1-MMP^Δ/Δ^ animals as compared to controls, while equal numbers of traced neuroblasts are observed in the proximal or intermediate part of the RMS (Fig. 4I). All in all, our data show that different models for the ablation of MT1-MMP (but not MT1-MMP expressing controls) have reduced numbers of olfactory glomeruli and exhibit decreased glomerular size in the OB. Consequently, MT1-MMP deficient mice have reduced numbers of periglomerular neurons. We interpret that increased cell-death rates in the distal RMS can adjust for the reduced numbers of prospective periglomerular neurons that are destined towards the OB of MT1-MMP deficient animals.

## Discussion

We present here a model with a specifically reduced number of olfactory glomeruli in the absence of inflammation or developmental aberrations in the brain. A decrease in glomeruli was consistent with reduced numbers of newly generated periglomerular cells and reduced numbers of periglomerular neuronal subtypes. Interestingly, the reduction in new periglomerular cells is specifically associated with increased cell death-levels within the distal RMS, but not with other abnormalities that could potentially affect the supply of new interneurons to the glomerular cell layer like e.g. altered proliferation in the RMS or altered NPC turnover in the SVZ. Our data are consistent with the notion that the distal RMS has a specific role in controlling periglomerular cell-fate. Accelerated cell-death rates in the distal RMS of MT1-MMP deficient mice can adapt the number of newly generated periglomerular cells to the reduced demand for this cell-type in the OB. Investigating the relation between the extent of periglomerular neurogenesis and NPC turnover in the distal RMS irrespective of an altered contribution of subventricular neurogenesis was only feasible in a non-invasive model and by a detailed inspection of the numbers of proliferating, migrating and dying cells in the SVZ, RMS and OB neurogenic axis.

Olfactory sensation is initiated by odorant receptors expressed by olfactory sensory neurons, which are part of the olfactory epithelium that is located in the posterior nasal cavity^36^. The olfactory sensory neurons traverse the cribriform plate and innervate mitral cells in the OB, which carry the olfactory information to higher brain centres. Numerous olfactory sensory neurons and a small number of mitral cells form the glomeruli^36^. Each glomerulus is activated by a small range of odours and odour-dependent glomerular activity has been mapped in OB-areas. However, processing of olfactory stimuli does not seem to be organized in a straight-forward topographical map of glomeruli in the OB. In addition to the representation of odours by mitral neuronal-activity there seem to be other levels of information-processing required, which are contributed by a network of interneurons in the granule cell layer and in the glomerular layer. A further level of plasticity to the olfactory neuronal system is added by newborn interneurons which insert either into the granule cell- or the glomerular-layer^29^,^30^,^36^. Several independent studies investigated the role of the SVZ and the RMS in controlling periglomerular cell numbers^6^,^7^,^8^. However, the use of intracerebral injections^6^,^9^, cerebral lesions^7^ or acute chemical ablation of the olfactory epithelium^9^ likely generate pathological stimuli for subventricular neurogenesis^37^ which complicate the study of new RMS-derived neurons. To interpret the contribution of the SVZ or the RMS to new periglomerular neurons we took advantage of a model (MT1-MMP deficient mice) producing reduced numbers of glomeruli and reduced OB-size by a developmental process outside the brain. Mice lacking MT1-MMP expression (as compared to controls) exhibited a reduced complexity in the folding of the nasal turbinates, which necessarily decreases the overall surface of the olfactory epithelium and thereby diminishes the number of olfactory sensory neurons, which vitally contribute to the formation of olfactory glomeruli. Importantly, this approach causes a reduction in glomerular number without generating a wave of cell-death that can represent a neurogenic stimulus for by the SVZ^37^. We found, by BrdU-tracking, that reduced numbers of newborn cells associate with the glomeruli in MT1-MMP deficient mice in comparison to control animals. Tracking of BrdU-label retaining cells indicated no differences in all other layers of the OB with all models. If reduced numbers of new neurons arrive at their periglomerular destination this can be caused by decreased neurogenesis, slowed migration or increased cell-death. By quantification of the proliferation marker Ki67 and by three different BrdU-labelling protocols we found no indication for altered proliferation-rates in the SVZ or the RMS including the OB. Also, total cell numbers in these regions were not changed. The inspection of dividing cells (as well as the subsequent quantification of cell-death levels) was performed in a sub-region specific manner taking into account that different subsets of olfactory interneurons origin from different parts of the SVZ^30^. Next, we investigated the RMS morphology and potential differences in migration of NPCs. Given the continuous production of new NPCs in the SVZ in all experimental paradigms it can be concluded that arrested migration of NPCs should be visible as an enlargement in RMS size^10^. However, the morphology of the RMS proximal to the SVZ and the intermediate part of the RMS remained similar in all models (BrdU-tracking of DCX-labelled cells in the distal RMS even revealed a reduction of neuroblast cell numbers in MT1-MMP deficient animals). Also, a combined alteration in proliferative and migratory behaviour of NPCs can be excluded as this would have been detected by altered cell-division levels in short-term or long-term BrdU labelling or Ki67 immunohistochemistry.

After ruling out that any statistically significant effects in proliferation or migration of NPCs in the SVZ or large parts of the RMS can be detected by comparing our animal models we studied cell death-levels in the olfactory neurogenic axis. Therefore we performed *in situ* TUNEL-labelling, which visualizes DNA strand-breaks and indicates necrotic and apoptotic cell-death^38^. TUNEL-assays are highly reliable indicators of dying cells as they show the final stage of different cell-death programs. We found that the number of TUNEL+ cells was specifically and statistically significantly increased in the distal RMS, but not in any other region (including any other sub-region) in the SVZ or the RMS. In synopsis, our intervention-free model for reduced glomerular neurogenesis showed an adaptive response to the decreased demand for new periglomerular neurons only in one part of the SVZ - RMS - OB axis: We detected accelerated cell-death rates and reduced neuroblast cell numbers in the distal RMS. Thereby, our study supports previous reports, which used interventional techniques to show that the distal RMS has a role in generating new neurons for the glomerular layer. Furthermore, our study suggests a signalling mechanism between the glomerular layer of the OB and the distal RMS, which can relay information on a reduced demand for periglomerular neurons to the germinative centre in the RMS. To uncover the mechanisms regulating neural stem and precursor cell plasticity is of central interest for brain stem cell biology^39^ and we showed that a - yet unidentified - signalling cue between the OB and RMS can exist, which regulates cell-death levels in the putative RMS stem cell niche. Previous studies showed that adult neurogenesis in the dentate gyrus can be controlled by environmental factors^40^,^41^,^42^. The signalling cues promoting hippocampal neurogenesis and plasticity include neuronal input to the dentate gyrus^43^,^44^,^45^. In analogy to the work on hippocampal neurogenesis, studies have been performed to modify neurogenesis in the SVZ^13^,^46^ and it was shown that environmental factors (odorant enrichment) can transiently increase SVZ-proliferation^9^. However, it was also proposed that the basal level of SVZ proliferation and neurogenesis is not affected by altered sensory stimulation^10^. Our data support the notion that neurogenesis in the RMS is regulated independently from NPC-turnover in the SVZ^6^,^9^ and we show an adaptive response of the neurogenic region within the RMS to the reduced need for new periglomerular neurons.

## Additional Information

**How to cite this article**: Langenfurth, A. *et al*. Decreased demand for olfactory periglomerular cells impacts on neural precursor cell viability in the rostral migratory stream. *Sci. Rep*. **6**, 32203; doi: 10.1038/srep32203 (2016).

## Supplementary Material

Supplementary Information
