# Supplementary material for: Decreased demand for olfactory periglomerular cells impacts on neural precursor cell viability in the rostral migratory stream
Source: Sci Rep. 2016 Aug 30;6:32203. doi: 10.1038/srep32203 (PMC5004164; doi:10.1038/srep32203)
Supplement: Supplementary Information [file srep32203-s1.pdf]

Supplementary information for the study entitled: Decreased demand for olfactory periglomerular cells impacts on neural precursor cell viability in the rostral migratory stream.

Anika Langenfurth[1,2,+], Song Gu[3,4,+], Verena Bautze[5]; Caiyi Zhang[3], Julia E. Neumann[6], Ulrich Schüller[6], Kristin Stock[1], Susanne A. Wolf [1], Anna-Maria Maier[5], Giorgia Mastrella[3], Andrew Pak[7], Hongwei Cheng[4], Roland E. Kälin[3], Kenn Holmbeck[7], Jörg Strotmann[5], Helmut Kettenmann[1] and Rainer Glass[3,*]

[1]Cellular Neurosciences, Max Delbrück Centre for Molecular Medicine, Robert Rössle Str. 10, Berlin, Germany;

[2]Department of Psychiatry and Psychotherapy/ Neuropsychiatry, Charité - Universitätsmedizin Berlin, Charitéplatz 1, Berlin, Germany;

[3]Neurosurgical Research, Ludwig Maximilians University Munich, Marchioninistr. 15, Munich, Germany;

[4]Department of Neurosurgery, The First Affiliated Hospital of Anhui Medical University, Hefei, Anhui, China, 230022

[5]Institute for Physiology, University of Hohenheim, Garbenstr. 30, Hohenheim, Germany;

[6]Center for Neuropathology, Ludwig Maximilians University Munich, Feodor-Lynen-Str. 23, Munich, Germany;

[7]Craniofacial and Skeletal Diseases Branch, NIDCR, National Institutes of Health (NIH), Bethesda, Maryland 20892-4380, USA.

+These researchers contributed equally

*Author for correspondence: Rainer Glass, PI Neurosurgical Research, Ludwig Maximilians University Munich, Marchioninistr. 15, 81377 München, Germany, rainer.glass@med.uni-muenchen.de

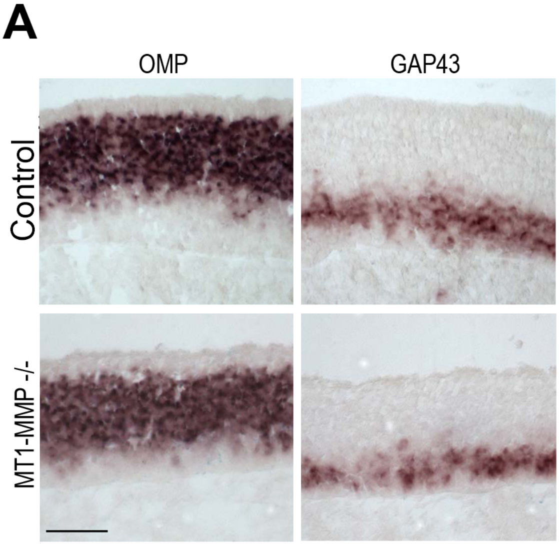

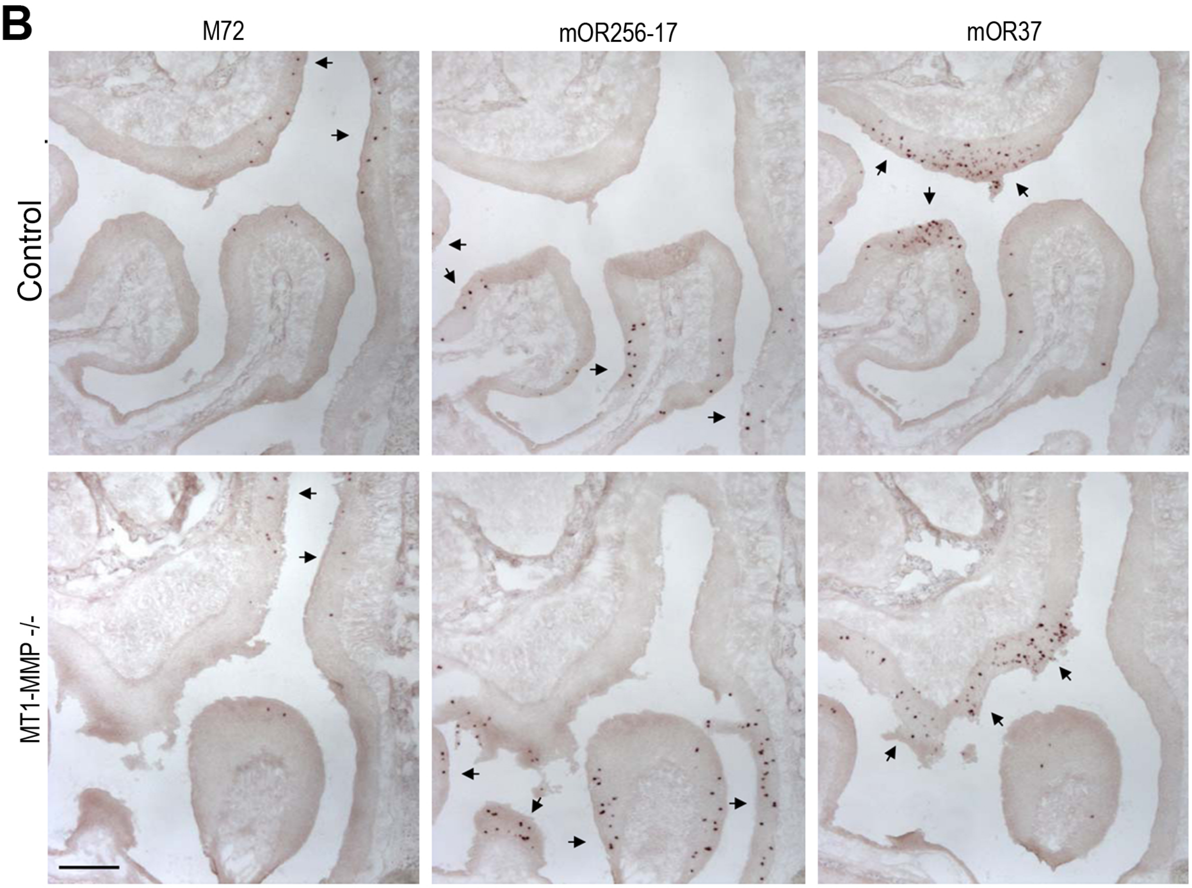

Langenfurth et al., supplemental Figure 1

Supplemental figure 1. Differentiation and localisation of olfactory sensory neurons are not affected by MT1-MMP expression. (A) Representative cross sections through the olfactory epithelium of MT1-MMP knock-out animals and MT1-MMP heterozygous animals (which phenotypically correspond to the MT1-MMP wild type) at postnatal day P20. Sections were hybridized with antisense riboprobes specific for the olfactory marker protein (OMP) or for a marker for immature olfactory sensory neurons (GAP43). OMP-positive (mature) and GAP43-positive olfactory sensory neurons were organized in the same manner in both genotypes. **(B)** Sections were hybridized with antisense riboprobes specific for the odorant receptors (ORs) M72, mOR256-17 and mOR37A. Arrows indicate the characteristic locations of OSNs which express these OR genes in distinct areas of the OE. Scale bar: Scale bars: 50 µm in A; 200 µm in B.

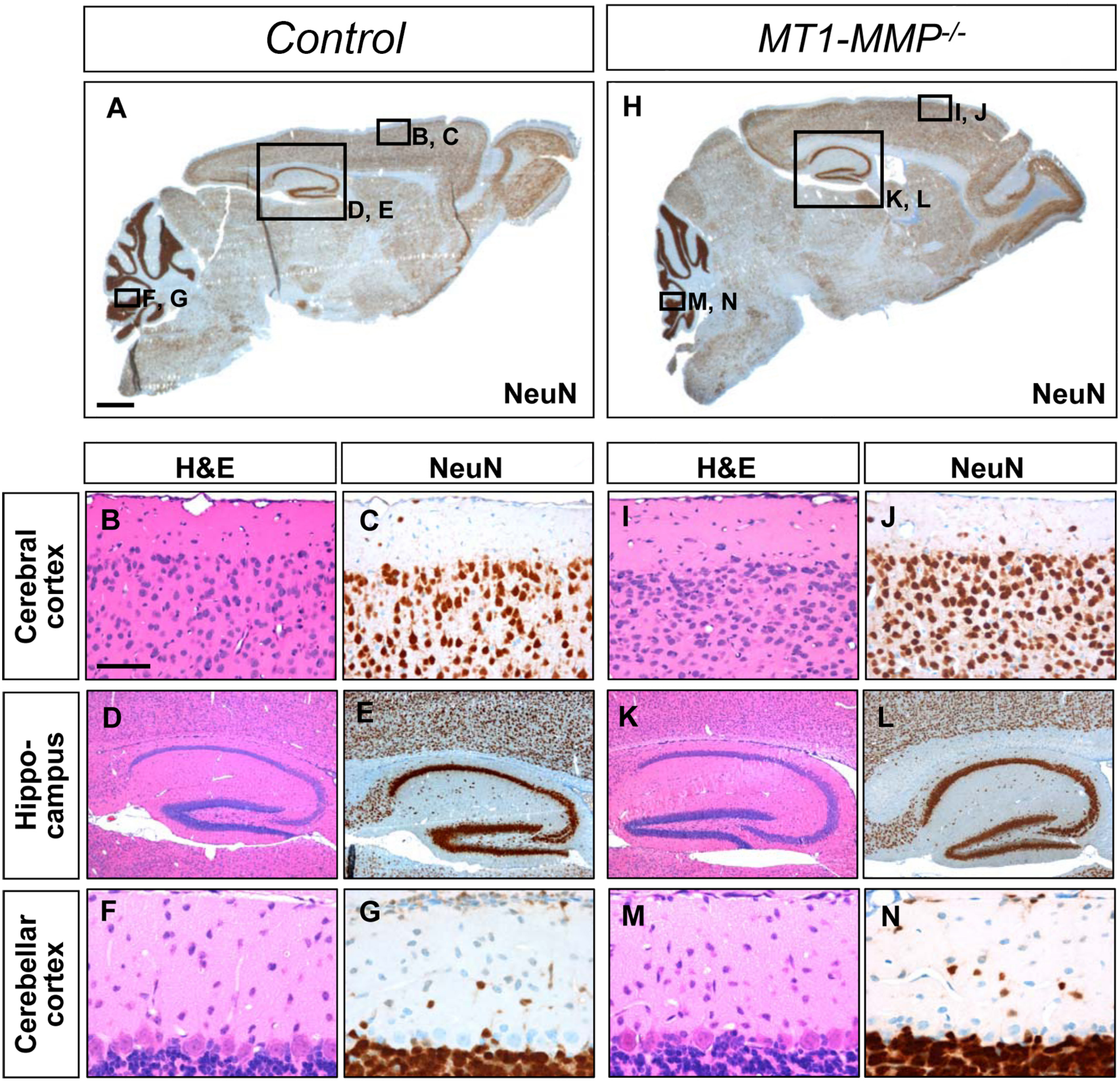

Langenfurth et al., supplemental Figure 2

Supplemental figure 2: MT1-MMP-/-mice showed inconspicuous CNS-histomorphology outside the olfactory bulb. **(A, B)** Sagittal sections of adult brains of *MT1-MMP*$^{+/+}$ (controls) and *MT1-MMP*$^{-/-}$ mice. Overall brain morphology did not reveal significant differences in *MT1-MMP*$^{-/-}$ mice when compared to controls. NeuN staining showed a physiological pattern of neuronal populations within the cerebral and cerebellar cortex, the basal ganglia and brainstem. **(B, C, I, J)** High power images of the cerebral cortex with upper isocortical layers (I-IV) showed adequate isocortical layering in *MT1-MMP*$^{-/-}$ mice. **(D, E, K, L)** Overall hippocampal morphology showed proper formation of CA1-4 regions and the dentate gyrus. **(F, G, M, N)** In the cerebellar cortex, the characteristic layering of molecular layer, Purkinje cell layer and internal granule cell layer did not show any abnormalities in H&E and NeuN staining. Scale bar in A is 1 mm for A and B. Scale bar in B is 100 µmfor B, C, I, J, 500 µmfor D, E, K, L and 50 µmfor F, G, M, N.

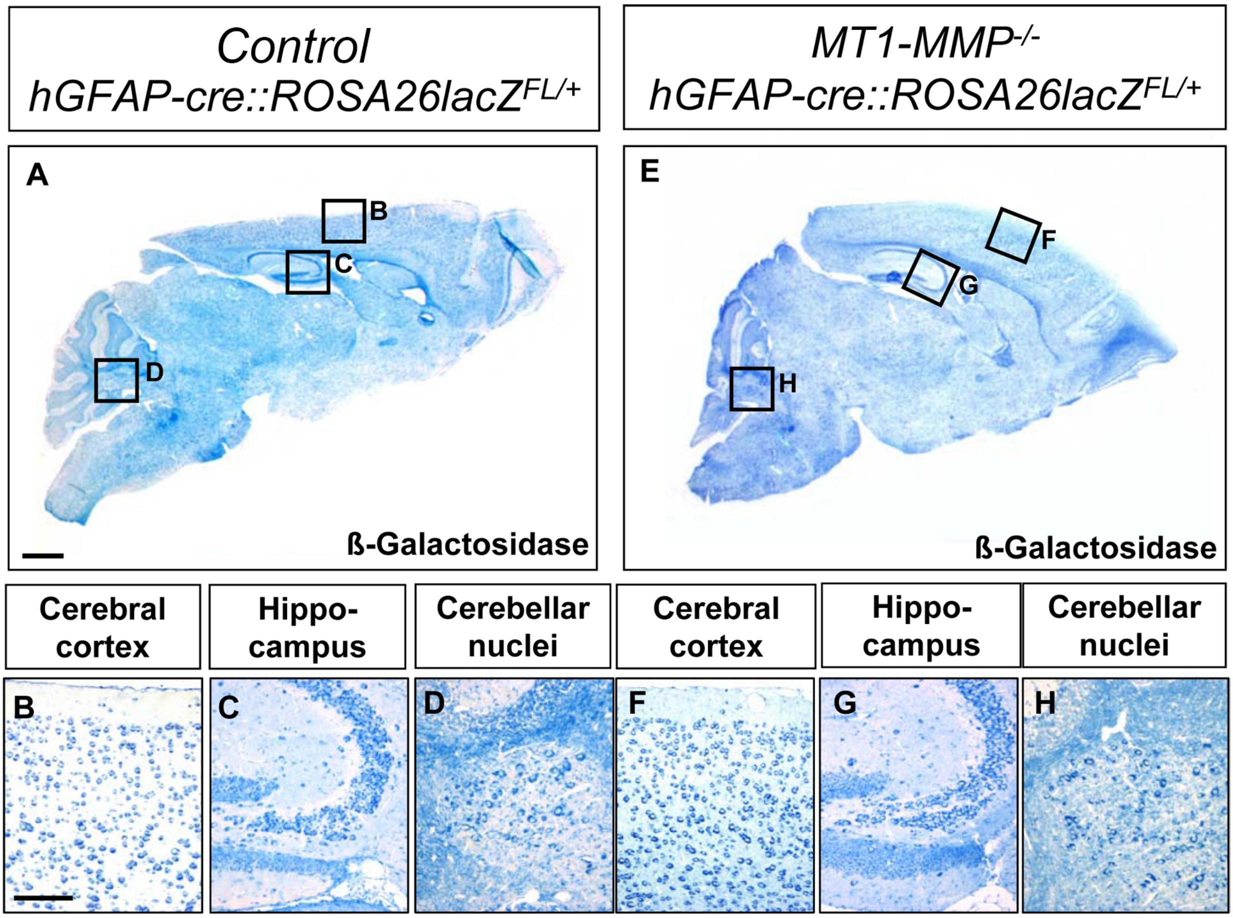

Langenfurth et al., supplemental Figure 3

Supplemental figure 3: Neural precursor cells generate identical cell populations in MT1-MMP deficient or wilde type mice. (A, E) Sagittal sections of adult brains of *MT1-MMP*$^{+/+}$*hGFAP-cre::ROSA26lacZ*$^{FL/+}$ (controls) and *MT1-MMP*$^{-/-}$*hGFAP-cre::ROSA26lacZ*$^{FL/+}$ mice. Immunohistochemistry using antibodies against ß-Galactosidase fatemapped cell populations all over the CNS in similar patterns. **(B, F)** *hGFAP*-positive precursors gave rise to neurons in all 6 layers of the cerebral cortex. **(C, G)** Neurons of the CA1 and 2 regions were intensely fatemapped in controls and in knockout mice. **(D, H)**The cerebellar nuclei were strongly fate mapped and did not display differences in controls and knockout mice. Scale bar in A is 1 mm (also representative for E). Scale bar in B is 200 µm for (also representative for C, D, F, G and H).

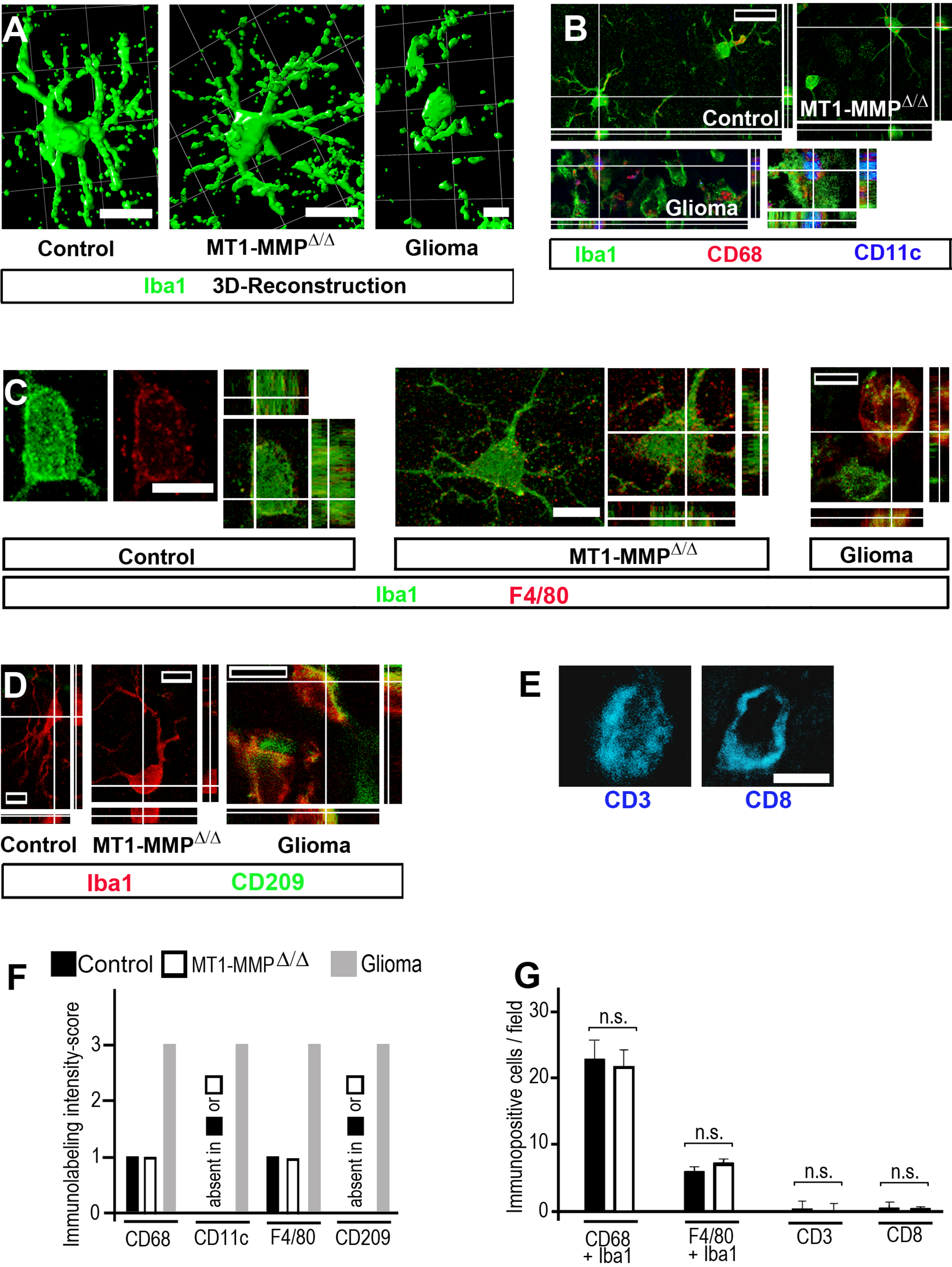

Langenfurth et al. supplemental Fig. 4

Supplemental figure 4: MT1-MMP deficient mice do not show any signs of neuro-inflammation. We investigated a range of immune-markers faithfully indicating microglia (Iba1 or CD68) and markers informing on functional states in myeloid cells (CD11c, CD209 or F4/80). In addition we performed immunofluorescence detection of lymphocytes in the CNS with antibodies for CD3 or CD8. Furthermore, we studied the three-dimensional cyto-architecture of microglia, as microglial cells undergo profound morphological changes during activation (see Hanisch UK & Kettenmann H. Nat Neurosci. 2007;11:1387-94.). We found that expression levels for some immune markers in both MT1-MMP-deficient and WT mice was very weak (F4/80) or even absent (CD11c or CD209), supporting the view that MT1-MMP-knockouts have no inflammatory reactions in the brain. In order to corroborate the validity of our immunolabeling paradigms we included samples from an orthotopic brain tumor (glioma) mouse-model: Gliomas contain large numbers of myeloid cells, tumor-associated myeloid-cells (TAM) display a very wide spectrum of immune markers and individual TAM can express ligands or receptors of the innate or adaptive immune response at high level. (**A**) 3-Dimensional reconstruction of large image-stacks from confocal microscopy revealed the typical ramified morphology of Iba1-labeled microglia (indicating a functional, immune-observatory state in the non-inflamed brain) in wild-type (control) and MT1-MMP-deficient (MT1-MMP$^{\Delta/\Delta}$) mouse brains; while TAM display an amoeboid morphology (that is acquired during M1 or M2 states of immune-activation; see e.g. Glass R, Synowitz M. Acta Neuropathol. 2014 128(3):347-62). (**B**) Confocal micrographs (showing a single optical section and the Z-axis of a 4 to 7µm stack) giving representative examples of immuno-labeling for Iba1 (green), CD68 (red) and CD11c (blue); note that CD11c (which has a role in the complement system and constitutes a marker for antigen presenting cells) is absent in controls or samples from MT1-MMP$^{\Delta/\Delta}$mice, but is abundant in TAM (one cell indicated by the cross-hair in the glioma-figure was shown as an enlarged figure; the blue-channel was shifted towards light-blue in order to increase visibility). (**C**) Immunofluorescence detection of Iba1 (green) and the F4/80 antigen (which is upregulated in inflammatory events, e.g. during neurodegeneration) is weakly expressed in control (single channel labelling and confocal colocalization-analysis are shown) or MT1-MMP$^{\Delta/\Delta}$samples (presenting a maximum-projection of a 5 µm image stack and a magnified optical section with corresponding Z-axis for the visualization of colocalizing pixels), but is intensely labeled in TAM (glioma). (**D**) The C-type lectin receptor CD209 is a marker for aberrant (M2-shifted) immune activation of microglia in neurodegenerative disease. Labelling for CD209 (in red) is intense in TAM but absent in control or MT1-MMP$^{\Delta/\Delta}$brain sections. (**E**) Immunofluorescence-labelling for CD3 or CD8 and confocal microscopy were used to visualize lymphocytes; image stacks (Z-axis is 3µm) are presented as maximum-projection; labeling for CD3 or CD8 was extremely rare in brain samples from control or MT1-MMP$^{\Delta/\Delta}$mice and was confined to the perivascular space. (**F**) Labeling-intensity for the set of immune markers described above was scored (0 = no labeling; 1 = weak; 2 = intermediate; 3 = intense) by confocal microscopy; therefore, identical

settings for microscopy recordings were used for each marker and fluorescence raw intensity values were obtained. (**G**) Immunofluorescently labeled cells were quantified from n = 4 animals per group, 6 sections per animal (horizontal brain sections with fixed stereotactical coordinates in the dorso-ventral axis) and 5 sampling sites per section (in the olfactory bulb, SVZ, cortex, striatum and hippocampus). Scale bar in A, C and D is 10µm; scale bar in B is 20µm; scale bar in E is 5µm; statistical significance in G is indicated as n.s. = not significant.

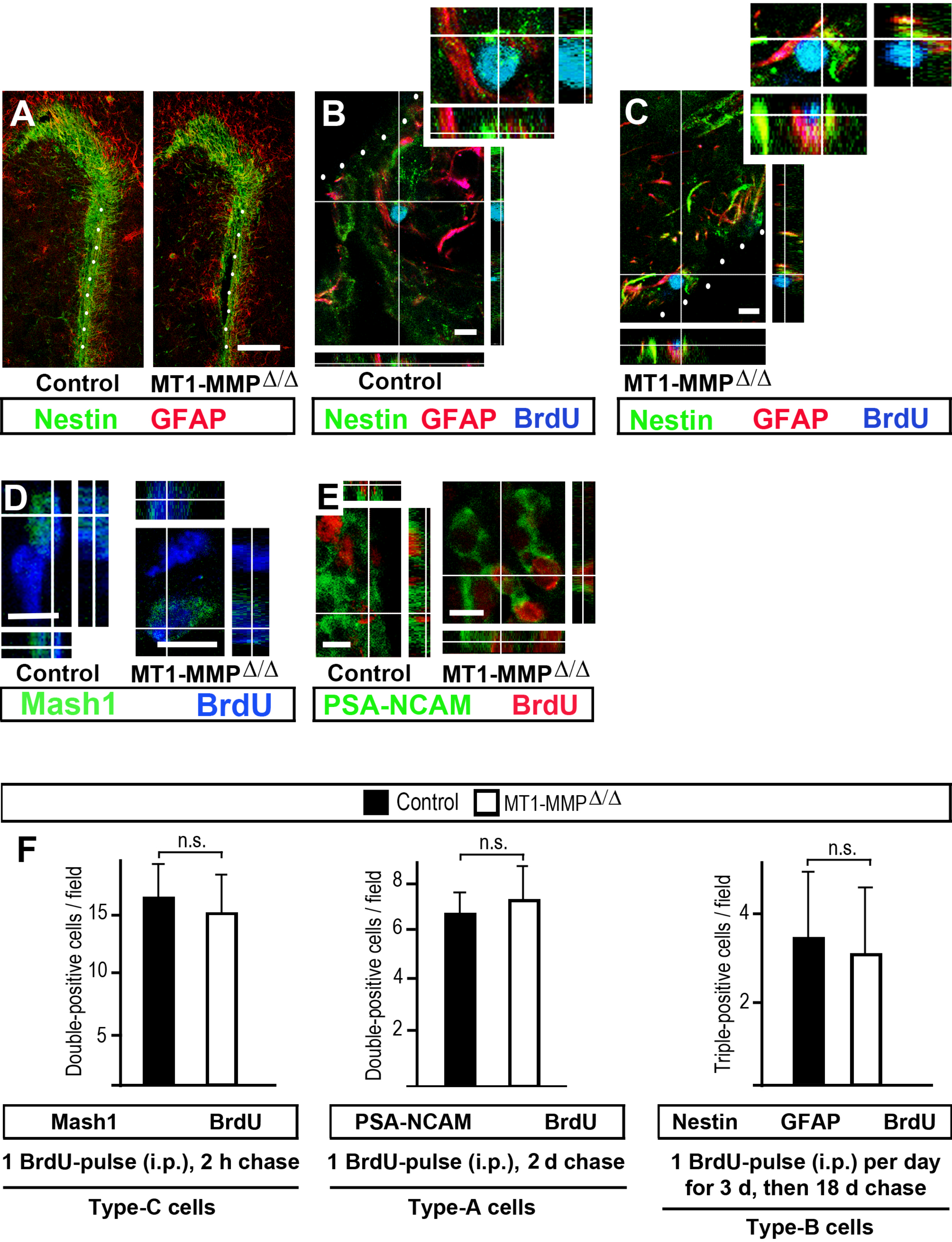

Langenfurth et al. supplemental Fig. 5

Supplemental figure 5: The contribution of distinct NPC subtypes to subventricular proliferation is not altered by MT1-MMP expression. (A) Immunofluorescence labeling for nestin (green) and GFAP (red) and subsequent confocal microscopy at low magnification provides an overview on the morphology of the SVZ in wild-type (control) and MT1-MMP-deficient (MT1-MMP$^{\Delta/\Delta}$) mice; a horizontal brain section was used to image the SVZ in the left brain hemisphere and the narrow ventricular lumen is indicated by a dotted line; note that the morphology and marker expression appear highly similar in both groups. (**B** and **C**) Control and MT1-MMP$^{\Delta/\Delta}$ mice were intraperitoneally (i.p.) injected with BrdU (1 BrdU pulse per day for 3 consecutive days) followed by an 18 days chase period; then mouse brains were immunolabeled for GFAP (red), nestin (green) and BrdU (blue) and inspected by confocal microscopy at high magnification to locate cells with colocalizing pixels. An overview on the immunofluorescence staining is shown in single optical sections and corresponding fluorescence-signal in Z-orientation is presented; single cells outlined by cross-hairs are magnified and marker colocalization is demonstrated; the lumen of the lateral ventricle is indicated by a dotted line; note that the BrdU labeling paradigm is highly specific for GFAP and nestin coexpressing subventricular (type-B) cells (blue labeling was shifted to light-blue for improved visualization). (**D**) Control and MT1-MMP$^{\Delta/\Delta}$ mice were i.p. injected with BrdU (1 BrdU 2h prior to sacrifice; labeling rapidly proliferating type-C cells) and tissue was immunolabeled for Mash1 (blue) and BrdU (red); single optical sections from confocal microscopy and corresponding immunofluorescent labeling in Z-direction is shown; note that Mash1-positive (type-C) cells abundantly label for BrdU. (**E**) To investigate dividing type-A cells we used another BrdU-labeling paradigm (1 BrdU-pulse i.p. followed by a 2 days chase period) in control and MT1-MMP$^{\Delta/\Delta}$mice; then mouse brains were immunolabeled for PSA-NCAM (green) and BrdU (red) followed by confocal microscopy; single optical sections and corresponding immunofluorescence in Z-orientation is shown; note that type-A cells (PSA-NCAM-positive cells) in both groups of mice are in the cell-cycle (BrdU-positive). (**F**) Immunofluorescence-marker positive cells from the experiments described in (B) through (E) were quantified using material from n = 4 animals per group, 6 sections per animal (horizontal brain sections with fixed stereotactical coordinates in the dorso-ventral axis) and 3 sampling sites in the SVZ (frontal to caudal axis). Scale bar in A is 200 µm, scale bars in B through E are 10µm; statistical significance in F is indicated as n.s. = not significant.

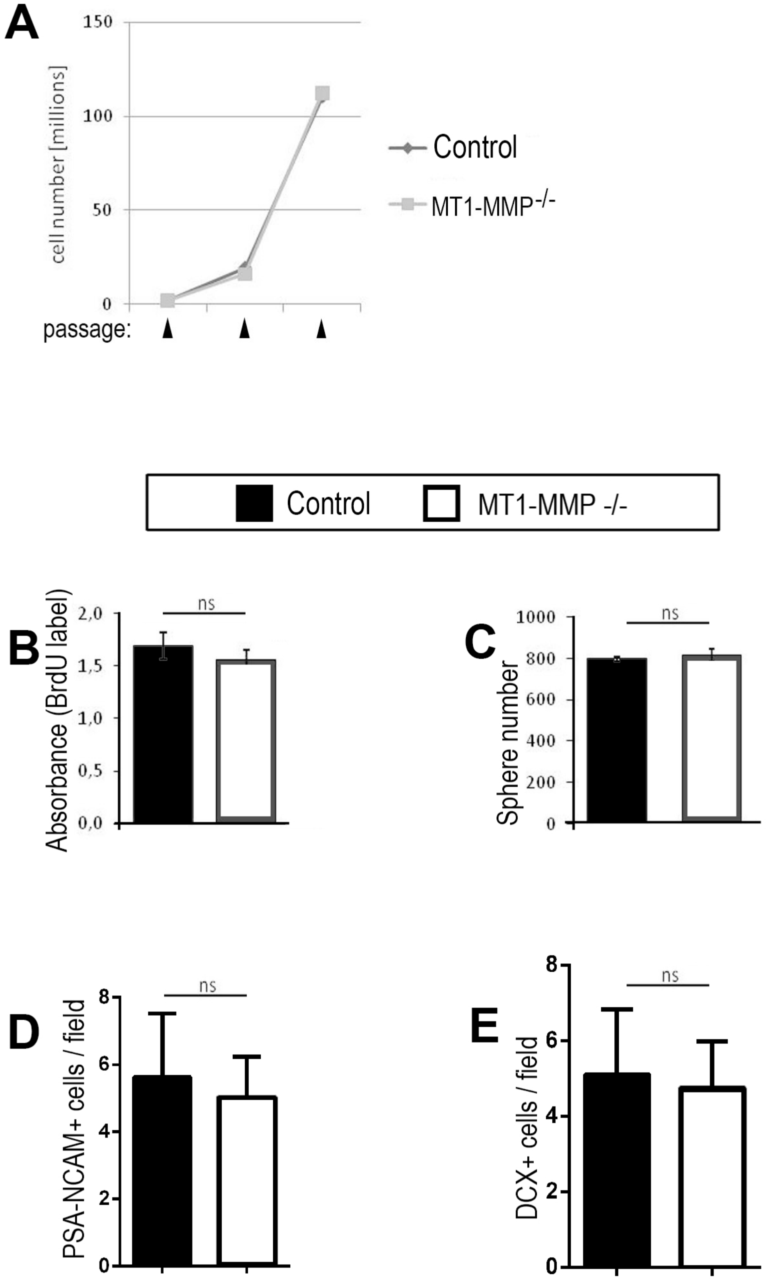

Langenfurth et al., supplemental Figure 6.

Supplemental figure 6: *In vitro* analysis of subventricular neural precursor cells from MT1-MMP$^{-/-}$ and controls. Cultivated neural precursor cells from P14 SVZ of MT1-MMP$^{-/-}$ and from controls had similar doubling times **(A)**, BrdU levels **(B)** and sphere formation capacity **(C)**. (**D** and **E**) Furthermore we investigated the potential of NPCs from MT1-MMP$^{-/-}$ and control mice for differentiation into the neuronal lineage. Therefore, NPC-cultures were maintained without growth factors and supplemented with 0.5% fetal calf serum; after seven days adherent cells were fixed, immunostained for PSA-NCAM or DCX and quantified. Statistical significance in B through E is indicated as n.s. = not significant.
